# Co-assembling de novo designed peptide with high-payload drug protein for noninvasive treatment of corneal neovascularization

**DOI:** 10.1016/j.ijpx.2025.100410

**Published:** 2025-09-28

**Authors:** Yuhua Tong, Sijie Zhou, Yongjie Guo, Xiaoli Jin, Meiting Yu, Chunyun Feng, Hao Chen, Xingjie Zan, Jinyang Li

**Affiliations:** a National Clinical Research Center for Ocular Diseases, Eye Hospital, Wenzhou Medical University, Wenzhou 325027, China; b Department of Ophthalmology, The Quzhou Affiliated Hospital of Wenzhou Medical University, Quzhou People's Hospital, Quzhou, 324000 Zhejiang, China; c Department of Ophthalmology, Second Affiliated Hospital of Chongqing Medical University, 400010 Chongqing, China; d No. 906 Hospital of People's Liberation Army, Wenzhou 325000, Zhejiang, China; e National Engineering Research Center of Ophthalmology and Optometry, Eye Hospital, Wenzhou Medical University, Wenzhou 325027, China; f State Key Laboratory of Eye Health, Eye Hospital, Wenzhou Medical University, Wenzhou 325027, China; g Wenzhou Institute, University of Chinese Academy of Sciences, Wenzhou 325000, Zhejiang, China

**Keywords:** Protein delivery, Peptide assembly, Protein drug, Corneal neovascularization, Nanomedicine

## Abstract

The specificity and low toxicity of protein drugs are significant for disease treatment but are strongly limited by their weak tissue penetrative capacity. Although formulating proteins with nanoparticle is an alternative strategy, the low encapsulation efficiency (EE) and loading capacity (LC) of protein drugs and their potential for protein inactivation remain significant challenges. Herein, the de novo designed peptide (Arg-His-Cys-Arg-His-Cys-Arg-His-Cys) (RHC)_3_, zinc ions (Zn^2+^), and the anti-neovascular protein drug Bevacizumab (Beva) were co-assembled to form PZA@Beva (peptide and Zn^2+^ assemblies encaspsulated Beva) nanomedicine, aiming to overcome the challenges associated with corneal neovascularization (CNV) model. The optimized size of PZA@Beva is approximately 162.5 nm, with EE% and LC% of Beva 92.7 % and 55.8 %, respectively. The bioactivity of encapsulated Beva was preserved, protecting it from proteolytic degradation, and the release of Beva from PZA@Beva exhibited pH-dependent kinetics. In vitro, PZA@Beva demonstrated effective penetration across the ocular barrier via both the paracellular pathway (by opening corneal tight junctions) and the transcellular pathway (through rapid cellular endocytosis). Additionally, PZA@Beva exhibited no cytotoxicity in vitro or in vivo, coupled with prolonged ocular retention, collectively yielding promising results for the treatment of CNV. This study contributes to non-invasive protein delivery across ocular bio-barriers for the treatment of diseases in the anterior segment.

## Highlights

• PZA@Beva was formed by co-assembling (RHC)_3_, Zn^2+^, and Beva under mild conditions.
• PZA@Beva, about 162.5 nm, shows high loading capacity and encapsulation efficiency.
• It has long retention on the ocular surface and enhanced penetration ability.
• PZA@Beva showed excellent treatment in the corneal neovascularization model.

## Graphical abstract

**Figure:**
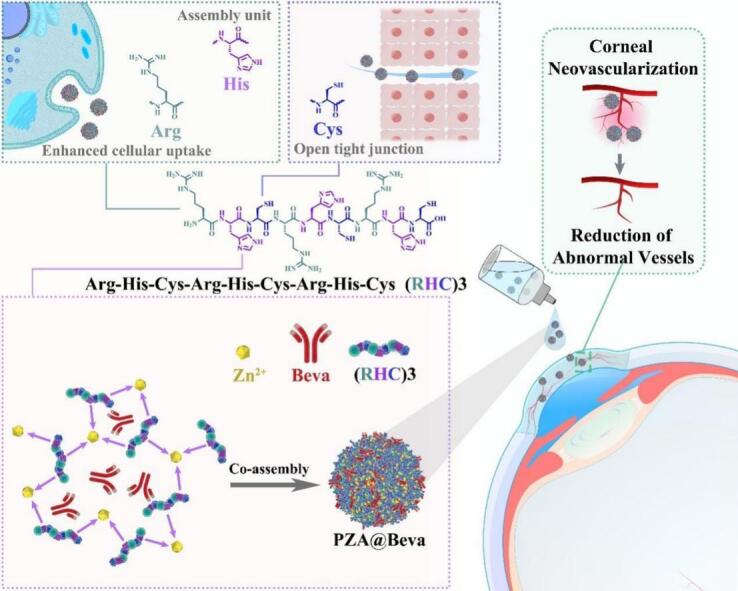

## 1 Introduction

Corneal neovascularization (CNV) is a prevalent ocular pathology characterized by the abnormal ingrowth of blood vessels into the avascular corneal stroma (Nicholas and Mysore, 2021a; Mitchell et al., 2018). The condition typically arises in response to various factors, including trauma, infection, inflammation, or hypoxia, and currently affects over a million individuals worldwide (Nicholas and Mysore, 2021b; Mitchell et al., 2018). Clinically, CNV is of significant concern due to its substantial impact on ocular health, as it compromises corneal transparency and disrupts normal visual processes (Hashemi et al., 2014). Protein-based therapeutics, such as bevacizumab (Beva), a monoclonal antibody targeting vascular endothelial growth factor (VEGF), have gained attention for their high specificity and low toxicity, making them promising candidates for CNV treatment (Aslan et al., 2023; Tomé et al., 2015). The presence of multiple biological barriers in the ocular complicates the effective delivery of protein-based therapeutics. To overcome these barriers and enhance bioavailability, invasive administration methods are often required. However, such approaches introduce risks of infection and may cause psychological distress in patients, limiting their acceptance and adherence to treatment.

Eye drops are a common and widely accepted route of administration for the treatment of ophthalmic diseases because of their excellent safety profile, high patient acceptance, and good therapeutic efficacy (Lakhani et al., 2018). The delivery of protein drugs via eye drops presents a significant challenge owing to the evolution of the eye over time, which results in the formation of multiple biological barriers that protect the body from foreign substances. These obstacles can be classified into two distinct categories: static and dynamic obstacles (Janagam et al., 2017). The static barrier is the network of tight junctions between epithelial cells, whereas the dynamic barrier is primarily the high blink frequency (3–5 s) and rapid replenishment of tear fluid (approximately ten minutes) on the ocular surface. Another challenge for protein delivery is the presence of protein hydrolases in the intraocular environment, which leads to inactivation of protein drugs. The presence of these biological barriers leads to low pharmaceutical efficacy when administering eye drops: less than 5 % for small molecules and nearly zero for proteins (Sikandar et al., 2011; Joseph and Venkatraman, 2017; Attia and MacKay, 2022).

The key to improving drug utilization is overcoming these barriers by prolonging drug retention on the ocular surface and enhancing the penetration ability of the drug, which can be achieved by formulating protein drugs with viscosity modifiers or penetration enhancers. Transepithelial permeation is characterized by two discrete modes of transport: the paracellular pathway, which occurs through intercellular tight junctions between cells, and the transcellular pathway, which requires cellular endocytosis (Hosoya et al., 2005). A growing body of research indicates that nano-delivery systems offer significant advantages in protein drug delivery across the ocular surface barrier, owing to their ability to alter the kinetics, intraocular distribution of protein drugs, and protection from protein hydrolases (Sahu et al., 2021). For example, Shen et al. used fluorocarbon chain-modified chitosan to open the tight binding barrier of the ocular surface to enable the delivery of protein drugs using eye drops (Shen et al., 2023). Yang et al. demonstrated ultrasmall micellar particles (approximately 10 nm) with high tissue permeability to deliver Beva via the transcytosis pathway in eye drops(Yang et al., 2024). However, encapsulating proteins into a delivery system presents a series of significant challenges (Yu et al., 2016). Perhaps the most critical issue pertains to the sensitivity of proteins to their microenvironments. The organic solvents and/or elevated temperatures employed in this encapsulation process, in conjunction with the hydrophobic microenvironment within the carrier itself, may potentially lead to the inactivation of proteins (Cao et al., 2019; Muheem et al., 2016). Moreover, encapsulation efficiency and loading capacity of protein-based pharmaceuticals in carriers are frequently not achieved, leading to suboptimal performance (McClements, 2018). The consequence of low encapsulation efficiency is wastage of expensive protein drugs, which results in increased costs. Moreover, low loading capacity impairs the carrier's ability to rapidly achieve a therapeutic concentration, which is crucial for the successful treatment of diseases (Chen et al., 2022). In addition, a low loading capacity can necessitate the use of excessive quantities of carrier material, which may present potential toxicity issues for the carriers (Luther et al., 2020).

**Scheme 1:**
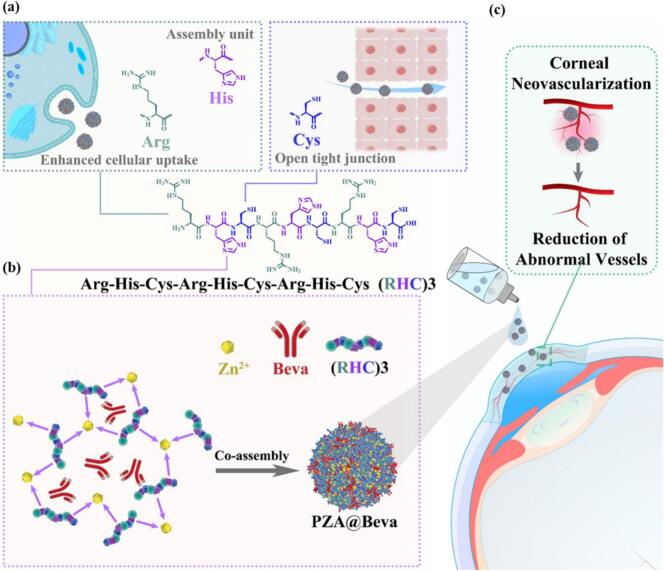
(a) The de novo designed peptide, Arg-His-Cys-Arg-His-Cys-Arg-His-Cys, (RHC)_3_, in which the histidine was employed for protein drug encapsulation assembly, the Cys for tight junction opening, and the Arg for enhanced cellular uptake; (b) The generation of PZA@Beva through co-assembling (RHC)_3_ and Beva in the presence of zinc ions; (c) The treatment of CNV by PZA@Beva.

Our laboratory has recently demonstrated that protein drugs can be packed into coordinate assemblies of hexahistidine and metal ions with effective encapsulation and high drug-loading capacity (Huang et al., 2019). Notably, this encapsulation process is conducted in a mild manner and the carrier microenvironment is highly hydrophilic, which restores the bioactivity of the encapsulated protein (Tang et al., 2022). In addition, this approach prevents proteins from being degraded by proteinases, confers the ability of protein drugs to penetrate cell membranes, and delivers proteins across the ocular surface barrier (Tang et al., 2022). Sulfhydryl-rich polymers have been reported to inhibit the tight junction closure through a glutathione (GSH)-mediated mechanism, thereby promoting the opening of epithelial tight junctions and increasing the permeability of drugs (Zhang et al., 2018; Armengol and Laffleur, 2021). Arginine-rich peptides enhance endocytosis by bonding guanidinium groups on arginine to phosphate and sulfate groups on the cell surface (Pescina et al., 2018). Herein, a novel peptide, Arg-His-Cys-Arg-His-Cys-Arg-His-Cys (RHC)_3_, was designed in which histidine was employed for protein drug encapsulation assembly, Cysteine (Cys) for tight junction opening, and Arginine (Arg) for enhanced cellular uptake (Scheme 1a). The (RHC)_3_ peptide and zinc ions (Zn^2+^) assemblies (PZA) were generated under pH around 8, and the PZA@Beva nanomedicine was formed by encapsulating Beva during the PZA assembly process (Scheme 1b). The resulting PZA@Beva displayed enhanced retention on the ocular surface and efficiently delivered Beva across the epithelial barriers via both paracellular and transcellular pathways, resulting in better therapeutic results than those of the subconjunctival injections frequently used in the clinic (Scheme 1c).

## 2 Materials and methods

### 2.1 Materials

Peptides, including Arg-His-Cys-Arg-His-Cys-Arg-His-Cys (RHC)_3_ and tetramethylrhodamine (TAMRA)-labeled (RHC)_3_ were synthesized by Nanjing Peptide Biotechnology Co., Ltd. (Nanjing, China). All the chemicals were of analytical grade. 4-(2-hydroxyethyl)-1-piperazineethanesulfonic acid (HEPES), polyvinylpyrrolidone (PVP, Mw ∼ 58 k, Sigma), zinc nitrate hexahydrate, hydrochloric acid, bovine serum albumin (BSA), ethylene eiamine tetraacetic acid (EDTA), sodium bicarbonate (NaHCO_3_), sodium carbonate (Na_2_CO_3_), ammonium chloride (NH_4_Cl), fluorescein isothiocyanate (FITC), bicinchoninic acid (BCA) kit, paraformaldehyde, Triton X-100 and sodium hydroxide were purchased from Aladdin (Shanghai, China). Beva was purchased by Roche (Swiss). All cell lines were purchased from the American Type Culture Collection (ATCC). The culture medium (Dulbecco’ s modified Eagle's medium, DMEM), fetal bovine serum (FBS), trypsin, and penicillin-streptomycin were purchased from GIBCO (California, America). Cell Counting Kit-8 (CCK-8) reagent was purchased from Vazyme (Nanjing, China). 3D multilayer corneal epithelial cells were purchased from Biocell Biotechnology Co., Ltd. The Corning matrix gel was purchase from Corning Co., Ltd. (America). Protease K, 4′,6-Diamino-2-phenylindole (DAPI) and Lyso-Tracker labeled with TRITC (Lyso-Tracker Red) were ordered from Beyotime. Beva ELISA kit, VEGF-A antibody, CD31 antibody and Occludin antibody were orderd from Abcam (UK).

### 2.2 Synthesis of PZA and PZA@Beva

#### 2.2.1 Synthesis and optimization of PZA and PZA@Beva

Under continuous ultrasonic conditions (80 KHz), Zn^2+^ was added dropwise to 1 mL of HEPES buffer (50 mM, pH 7–8) containing PVP (5 mg/mL) and (RHC)_3_ solution (4 mg/mL). Then, PZA nanoparticles were collected by centrifugation (4 °C, 12000 rpm, 15 min) after continuous ultrasound for 10 min and washed three times with deionized water. At a fixed concentration of (RHC)_3_ (4 mg/mL) in a 1 mL reaction system, the molar ratio of (RHC)_3_ to Zn^2+^ (10:1, 5:1, 2:1, 1:1, 1:2, 1:5, and 1:10) was adjusted by adding different amounts of Zn^2+^, and the nanoparticles at the optimal ratio were selected for subsequent experiments. Under the optimized conditions for fabricating PZA, a 500 μL system containing various masses of Beva (0.25, 0.5, 1, 1.5, 2, 2.5, and 3 mg) was employed to explore the optimized Beva concentration during entrapment. The PZA@Beva particles were generated using the same protocol as that used for PZA.

#### 2.2.2 The encapsulation efficiency of PZA@Beva

For ease to calculate the percentages of encapsulation efficiency (EE%) and loading capacity (LC%) of PZA@Beva, the Fluorescein isothiocyanat-labeled Beva (FITC-Beva) was applied. FITC-Beva was synthesized by adding 100 mg Beva to 2 mL NaHCO_3_/Na_2_CO_3_ (1:1 and 0.2 M for each) buffer (pH = 8, don't need pH adjustment), then adding 2 mg FITC and stirring overnight at 4 °C in dark environment, and adding NH_4_Cl (50 mM) to terminate the reaction. The solution was placed in a dialysis tube with a molecular weight cut-off of 3.5 KDa, and dialyzed in HEPES buffer (0.1 M, pH 7.2) for 4 days in the dark, during which the dialysate was changed at a frequency of 8 h. The solution was freeze-dried and the powder was stored at −20 °C.

EE% was calculated using FITC-Beva and a microplate reader according to the following formula:

$$\mathrm{EE}\%=\frac{V_{0}*C_{0}-V_{1}*C_{1}}{V_{0}*C_{0}}*100\%$$

Where V_0_ and V_1_ represent the volumes of the original solution and the supernatant solution, respectively, and C_0_ and C_1_ represent the concentration of FITC-Beva in the original solution and the supernatant solution, respectively. C_0_ and C_1_ were determined by the standard curve (Fig. S1), based on the UV–vis absorption intensity at 495 nm.

A certain mass of PZA@FITC-Beva was disassembled using EDTA (10 mM, 50 μl), and the concentration of Beva was quantified using a microplate reader according to the standard curve. LC% of PZA@Beva was calculated using the following formula:

$$\mathrm{LC}\%=\frac{W_{1}\text{of Beva}}{W_{2}\;\text{of}\;\mathrm{PZA}@\text{Beva}}=\frac{V_{x}*C_{x}}{W_{2}\;\text{of}\;\mathrm{PZA}@\text{Beva}}*100\%$$

Where W_1_ and W_2_ represent the weights of the tested Beva and added PZA@Beva, respectively, V_x_ is the volume of PZA@Beva disassembled in the EDTA solution, and C_x_ is the concentration of FITC-Beva determined by the standard curve (Fig. S1), based on the UV–vis absorption intensity at 495 nm.

#### 2.2.3 The release of PZA@Beva

5 mL PZA@Beva solution (Beva: 4 mg/mL) was added in a dialysis tube (Mw = 100KDa), and then the dialysis tube was placed in 45 mL Bistris buffer of different pH (pH = 5.8, 7.2) and shaken at a constant speed of 200 rpm in a shaker at 37 °C. One milliliter of the supernatant was removed at different time points during storage, and an equal amount of buffer of the same pH was immediately added. Finally, a bicinchoninic acid (BCA) kit was used for quantitative analysis of all supernatants to calculate the cumulative release amount at different time points, as previously reported (Davis et al., 2014).

#### 2.2.4 The bioactivity assay of Beva

EDTA (10 mM, 50 μl) was added to the PZA@Beva solution and vortex for 30 s to completely disassemble PZA and release Beva, and free Beva was used as the control. The bioactivity of Beva was tested using a Beva ELISA kit, with the following the protocol provided by the manufacture. Free Beva (control) and PZA@Beva co-incubated with Protease K (50 μg/mL) at 55 °C for 2 h. The bioactivity of Beva in PZA@Beva was tested using a Beva ELISA kit after releasing Beva from PZA@Beva by adding EDTA.

### 2.3 In vitro cellular assays

#### 2.3.1 Cell culture

Human corneal epithelial cells (HCECs) and human umbilical vein endothelial cells (HUVECs) were cultured in DMEM containing 10 % fetal bovine serum (FBS) and 1 % penicillin-streptomycin solution). All cells were incubated in a sterile incubator containing 5 % CO_2_ at 37 °C. And the following cellular experiments was the same protocol unless particularly mentioned. For 24-well and 96-well the volume of cell culture medium were 1 mL and 0.1 mL, respectively.

#### 2.3.2 Cytotoxicity test

HCECs and HUVECs were seeded in 96-well plates at a density of 5 × 10^3^ cells/well and incubated in an incubator for 24 h. After incubating the cells with a series of concentration of PZA or PZA@Beva (0, 20, 40, 60, 80, 100 μg/ml) for another 24 h, the culture medium was discarded and the CCK-8 reagent was added strictly according to the manufacturer’ s protocol to assess cytotoxicity.

#### 2.3.3 The cellular uptake of PZA@Beva

HCECs or HUVECs were seeded in 24-well plates at a density of 7 × 10^4^ cells/well and incubated in an incubator for 24 h. 500 μL culture medium containing free FITC-Beva (free drug, control) and PZA@FITC-Beva was added into each well. After 24 h, the medium in each well was discarded and the HCECs were washed three times with sterile PBS. HCECs or HUVECs were fixed with 4 % paraformaldehyde for 20 min, the cell nuclei were stained with DAPI, and lysosomes were stained with Lyso-Tracker labeled with TRITC and imaged under a Confocal Laser Scanning Microscope (CLSM, A1, Nikon). In subsequent cell experiments involving Beva, the concentration of Beva for all samples (Beva, PZA@FITC-Beva, or FITC-Beva) were maintained at 10 μg/mL unless specially mentioned.

#### 2.3.4 Effect of PZA@Beva on the distribution of occludin of HCECs

HCECs were seeded in 24-well plates at a density of 5 × 10^4^ cells/well and incubated in an incubator for 5 days. The culture medium was discarded and the medium contained Beva (free drug, control) or PZA@Beva. was added to the wells and incubated for 6 h. The HCECs were then washed three times with sterile PBS and fixed with 4 % paraformaldehyde at room temperature for 15 min. HCECs were permeabilized with 0.5 % Triton X-100 for 10 min and washed, followed by blocking of nonspecific proteins in the cells with 5 % BSA. HCECs were then incubated with an occludin monoclonal antibody for 3 h. Cell nuclei were stained with DAPI. All cells were imaged using a CLSM (A1, Nikon).

#### 2.3.5 Test of trans-epithelial electrical resistance (TEER) on the HCECs

The medium inside and outside of the blank Transwell plate was equilibrated to the same liquid level. The resistance of the blank Transwell was measured using Millicell ERS-2 after the medium was discarded. HCECs were inoculated into transwell plates at a density of 5 × 10^4^ cells/well. The chamber was placed in the corresponding well plate and cell culture medium was added to equalize the liquid levels inside (200 μL) and outside (800 μL) the chamber. The well plate was incubated at 37 °C in 5 % CO₂ for 5 days, with daily medium changes. On day 5, cell resistance was measured to assess the formation of tight junctions in HECEs. Once the resistance value exceeded 250 Ω/cm^2^, the medium inside the chamber was discarded, and medium containing Beva (free drug, control) or PZA@Beva was added for continued incubation. Cell resistance was measured and recorded every hour during incubation.

#### 2.3.6 The permeation test of PZA@Beva on the recombinant 3D multilayer corneal epithelial cells in vitro

3D multilayer corneal epithelial cells were cultured in sterile 6-well transwell plate. The culture medium containing FITC-Beva (free drug, control) or PZA@FITC-Beva was added to the upper chamber of the Transwell. The cells were incubated in the incubator for another 24 h. Then, the upper chambers of the Transwell were removed and washed three times with sterile PBS. Next, the cells were fixed with 4 % paraformaldehyde for 15 min and the nuclei were stained with DAPI. Finally, the membranes from the orifice plates were detached, observed, and photographed using a CLSM (A1, Nikon).

#### 2.3.7 Effect of PZA@Beva on the endothelial cell migration test

Briefly, seed human venous endothelial cells (HUVECs) in a 12-well plate at a density of 2*10^5^ cells/well and incubate them in the incubator for 24 h. Next, a sterile 200-μL pipette was used to make a scratch on the bottom of the cell-filled orifices, and the plates were gently washed with sterile PBS three times. A microscope was used to visualize the scratches. Culture medium containing Beva (free drug, control) or PZA@Beva was then added to the wells and incubated for 24 h. Scratch images were taken using a microscope after incubation and further analyzed using ImageJ software. The cell mobility (m) was calculated using the following equation:

$$m\left(\%\right)=\left(1-\frac{n}{r}\right)*100\%$$

where n represents the width of the scratches at 24 h and r represents the initial scratch width.

#### 2.3.8 Effect of PZA@Beva on the tubular formation test

50 μl of the Corning matrix was placed at the bottom of a 48-well plate and incubated in 37 °C incubator for 1 h to form a gel. HUVECs were seeded in the plate at a density of 5 × 10^4^ cells/well, and culture medium containing Beva (free drug, control) or PZA@Beva was added to the plates simultaneously. The plate was incubated for another 6 h. Then, the plate was imaged by CLSM (A1, Nikon) and further analyzed using ImageJ software.

#### 2.3.9 Lysosome escape test of PZA@FITC-Beva

HCECs were seeded in 24-well plates at a density of 5 × 10^4^ cells/well. After 24 h of incubation, the medium was replaced with 500 μL culture medium containing Beva (free drug, control) or PZA@FITC-Beva. After an additional 8-h incubation, lysosomal probes (Lyso-Tracker Red) were diluted in DMEM (1:20,000) and added to the wells. The plates were then incubated for 2 h in the dark. Nuclei were stained with DAPI, and an anti-fluorescence quencher (Thermo Fisher Scientific) was added. The sample was sealed with nail polish and imaged by CLSM (A1, Nikon). Colocalization results were analyzed using ImageJ software, and Pearson's coefficients were calculated. Lyso-Tracker Red and the anti-fluorescence quencher were used according to the manufacturer's protocol.

### 2.4 In vivo test

#### 2.4.1 Animals

Animal experiments were conducted on male Sprague-Dawley (SD) rats (male, weighing 150–180 g), and were approved by the Animal Experiment Committee of Wenzhou Chinese Academy of Sciences (Syxk 2021–0040). Rats were free to access food and water during the experimental period. The left eye of the rats was designated as the control and the right eye was designated as the test eye. Prior to in vivo tests, the anterior segment of the eyes was examined using a slit-lamp microscope to ensure the absence of any lesions in the anterior segment.

#### 2.4.2 Construction and treatment of alkali burn–induced CNV model

The CNV model induced by alkali burns was constructed according to literature (Tang et al., 2022). Briefly, after intraperitoneal injection anesthesia of rats with pentobarbital sodium (40 mg/kg), a 3 mm circular filter paper soaked in 1 M NaOH was placed on the center of the cornea for 40 s to establish the CNV model. Then, The filter paper was removed, and the conjunctival sac was rinsed with pre-cooled (4 °C) normal saline for approximately 2 min. Next, 20 μL of saline (NS group), free Beva, or PZA@Beva solution was locally administered thrice daily for 7 days. Additionally, one group of rats received subconjunctival injections of equal doses of free Beva. On days 3, 7, and 14 post-administration, with overdose of pentobarbital sodium (intraperitoneal injection, 100 mg/kg) before CNV examination, immunofluorescence staining, and histological analysis. In the following test, the Beva concentrations for all groups (Beva, FITC-Beva, PZA@Beva or PZA@FITC-Beva) kept the same, 2.5 mg/mL. And the anesthesia and euthanasia were the same procedures described here. 5 rats were used for each group.

#### 2.4.3 Evaluation of retention time of PZA@Beva on ocular surface

Twelve normal SD rats were randomly divided into two groups. After anesthesia, 20 μL of FITC-Beva (free drug, control) or PZA@FITC-Beva solutions were added to the eye surface of rats in each group. The head area of the rats was imaged and photographed using the multi-mode in vivo imaging system at different time points (0, 0.5, 1, 2, and 3 h), and fluorescence quantitative statistics were analyzed using the built-in software of the instrument.

#### 2.4.4 Therapeutic evaluation of PZA@Beva on CNV

The inflammatory index and CNV area were calculated as previous report (Tang et al., 2022). On the 7th and 14th days of the experiment, the rats were euthanized and the eyeballs were removed for fixation, embedding, sectioned for hematoxylin and eosin (H&E) staining, and photographed with a fluorescence microscope. Similarly, sections of the eyeballs were used for immunofluorescence staining of VEGF-A and CD31 according to a previous report (Tang et al., 2022) and finally photographed using confocal laser microscopy.

#### 2.4.5 The distribution of Beva in rat cornea

Rats were anesthetized and sacrificed 0.5, 6, and 24 h after the first dose. Subsequently, an incision was made in the eye and the lens was removed. The eyes were rapidly implanted in the optimal cutting temperature compound (OCT), cut into 5 μm-thick sections prior to staining, and pretreated with 1 % Triton and 5 % goat serum solution. Beva staining was performed using FITC-labeled goat anti-human immunoglobulin (H + L) (1:500). After further staining the nuclei with DAPI, images were taken using a CLSM (A1, Nikon).

#### 2.4.6 Long-term biosafety

After 30 days of continuous eye treatment, the anterior segment of the mouse eye was examined using a slit-lamp. The mice were euthanized and the cornea and retina were carefully isolated using a scalpel. The major organs (the heart, liver, spleen, and kidneys) were also harvested. All tissues, including the cornea, retina, and major organs, were sectioned and stained with hematoxylin and eosin.

### 2.5 Characterization and instruments

The size, polymer dispersity index (PDI) and zeta potential of PZA and PZA@Beva were measured using dynamic light scatter (DLS) instrument (Zetasizer Nano ZS Malvern Instruments). The encapsulation of Beva in PZA was detected using UV–vis spectroscopy (Perkin Elmer, Lambda 25, USA). The morphologies of PZA and PZA@Beva nanoparticles were observed and recorded using transmission electron microscopy (TEM, Talos F200S FEI). Laser confocal microscope was used to observe and photograph immunofluorescence sections of cells and corneal tissues (Nikon, Japan). CCK-8 results were detected using microplate reader (Thermo Fisher, USA). Cell scratch test, tube formation test and HE staining section were observed and photographed by inverted fluorescence microscope (Nikon, Japan). Ocular drug retention time in rats was photographed using a multi-modal in vivo imaging system (PekinElmer, USA). The anterior segment of the rat eye was observed and photographed using a slit lamp microscope (Mocular, China). The secondary structure of Beva was characterized by circular dichroism (CD) spectra were of in the range of 190–300 nm (Applied Photophysics Ltd., UK).

### 2.6 Statistical analysis

The Student's *t*-test was used to assess the significance of the mean difference between the two groups. Statistical significance was set at *P* < 0.05. The results are significant as follows: ∗P < 0.05, ∗∗*P* < 0.01, ∗∗∗*P* < 0.001, and **** *P* < 0.0001; “NS” indicates insignificance. All statistical analyses were performed using the SPSS software (IBM SPSS Statistics 22.0). All experiments were independently repeated at least three times and the reported values are the means of at least three independent replicates (*n* ≥ 3) with the standard error.

## 3 Results

### 3.1 Synthesis and optimization of PZA nanoparticles

**Fig. 1:**
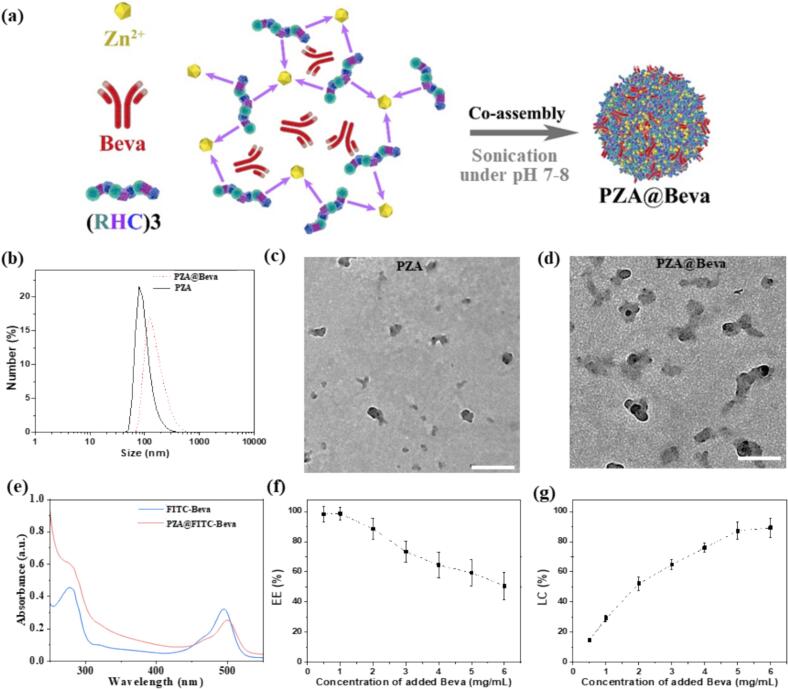
(a) Schematic illustration of the PZA synthesis. (b) The hydrodynamic size distribution of PZA and PZA@Beva, tested by DLS. (c-d) The TEM images of PZA and PZA@Beva. (e) UV–vis absorbance of free FITC-Beva and PZA@FITC-Beva. In 500 μL reaction system, with the molar ratio of Zn(NO_3_)_2_ to (RHC)_3_ at 1:2 and the concentration of (RHC)_3_ at 4 mg/mL, the dependence of (f) EE% and (g) LC% on the concentration of Beva during the generation of PZA@Beva. The PZA@Beva particles in b-e were generated under a 500 uL system containing 1 mg Beva /mL and 2 mg (RHC)_3_, with the molar ratio of Zn(NO_3_)_2_ to (RHC)_3_ at 1:2. The scale bar in (c) and (d) is 200 nm. Means (±S. E.) of *n* = 3 (at least) independent experiments.

In this study, PZA nanoparticles were synthesized using a one-step method, as illustrated in Fig. 1a. When Zn^2+^ was added to the (RHC)_3_ solution, the color of the solution changed from transparent to light blue, indicating the formation of the PZA nanoparticles (Fig. S2). To further optimize the PZA nanoparticles, in 500 μL system, the amount of (RHC)_3_ was fixed at 4 mg/mL, and the amount of Zn^2+^ was changed, and the formed particles were characterized by dynamic light scatter (DLS) instrument. As shown in Table 1, when the ratio of (RHC)_3_ to Zn^2+^ was 10:1, no distinct particle was formed in the solution. All PZA particles were positively charged. The charge increases as the amount of Zn^2+^ increases. When the ratio was 10:1, no nanoparticle was produced, which may be because the concentration of Zn^2+^ was too low to generate an effective coordination network with (RHC)_3_. As the concentration of Zn^2+^ increased, the particle size decreased with an increase in the polydispersity index (PDI) of the nanoparticles, until the ratio of (RHC)_3_: Zn^2+^ 1:5. At or after the (RHC)_3_: Zn^2+^ 1:5, the size and PDI increased with increasing Zn^2+^. It is possible that when excess zinc ions are added, they may form zinc hydroxide nanoparticle clusters under alkaline conditions, which could interfere with the coordination between zinc ions and histidine, ultimately affecting the particle size and PDI. At a (RHC)_3_: Zn^2+^ of 1: 2, the particle size and PDI of the nanoparticles reached a minimum, indicating that the nanoparticles were the smallest and most uniform. When the ratio was 1:2, the size and PDI of PZA were 104.2 nm and 0.21, and the zeta potential was +25.7 mV. Therefore, synthesis conditions with a concentration of (RHC)_3_ of 4 mg/mL and a ratio of (RHC)_3_: Zn^2+^ of 1:2 were selected for subsequent experiments.

**Table 1:** Effects of different molar ratio of (RHC)_3_: Zn^2+^ on size, PDI and zeta potential of PZA nanoparticles.

Ratio of (RHC)_3_: Zn^2+^	Size (nm)	PDI	Zeta potential (mV)
10:1	–	–	–
5:1	1585.01 ± 27.03	0.59 ± 0.12	6.71 ± 3.74
2:1	507.62 ± 17.40	0.40 ± 0.13	9.96 ± 3.18
1:1	429.58 ± 25.44	0.52 ± 0.11	21.3 ± 4.84
1:2	104.19 ± 5.49	0.21 ± 0.03	25.7 ± 3.99
1:5	243.45 ± 19.34	0.30 ± 0.12	27.1 ± 4.36
1:10	341.58 ± 24.39	0.44 ± 0.14	29.2 ± 3.67

Note: “-” means no particle was generated or the data was not detectable.

### 3.2 Encapsulation of Beva into PZA

Similarly, Zn^2+^ was added slowly to a solution of (RHC)_3_ and Beva, with (RHC)_3_: Zn^2+^ of 1: 2 and final pH of the formulation around 7.5. As shown in Fig. S2, the solution changed from transparent to light blue, indicating the formation of the PZA@Beva nanoparticles. Compared to PZA, the color of PZA@Beva was deeper, indicating more particles and/or larger particle sizes. The size of the PZA@Beva nanoparticles was determined using DLS. As shown in Fig. 1b, the size of PZA@Beva was 162.5 nm, slightly larger than PZA at 104.2 nm. TEM images revealed that both PZA (Fig. 1c) and PZA@Beva (Fig. 1d) exhibited uniform size and irregular morphology. No obvious aggregation was observed for either formulation, and both showed a narrow size distribution, consistent with the DLS results (Fig. 1b). To further verify the successful entrapment of Beva, UV spectroscopy was used to characterize the PZA@FITC-Beva. As shown in Fig. 1e, FITC-Beva had characteristic FITC and protein peaks at 495 and 280 nm, respectively. Similarly, FITC-Beva showed characteristic peaks in the corresponding regions after Beva entrapment, indicating that Beva was successfully encapsulated in PZA. EE% and LC% are two of the most important characteristics of drug-delivery vehicles. A high EE% indicates cost-effective conservation of protein drugs, whereas a high LC% makes therapeutic drug concentrations easier. Under optimized conditions, the EE% and LC% of Beva were tested by adding Beva at various concentrations (0.5, 1, 2, 3, 4, 5, and 6 mg/mL). When the Beva concentration was increased above 1 mg/mL, the EE% decreased with increasing Beva concentration (Fig. 1f), whereas LC% continued to increase with increasing concentration (Fig. 1g). As listed in Table S1, the size and PDI of PZA@Beva increased with concentration of Beva, while the zeta potential showed no obvious change. Considering these properties (size, PDI, zeta potential, EE% and LC% together, a Beva concentration of 2 mg/mL was selected as the optimal encapsulation concentration, at which the EE% was 92.7 % and LC% was 55.8 %. According to the report by Davis et al., the encapsulation efficiency of Beva in a liposome modified with annexin-5 was 25 % (Davis et al., 2014). Taheri et al. reported chitosan-*N*-acetyl-l-cysteine nanoparticles with an EE of 83 % (Taheri et al., 2022). The high EE% of PZA might be attributed to the multiple intermolecular interactions (electrostatic interaction between positively charged arginine or histidine and negatively charged Beva; bridge-connected coordinative interactions between Zn^2+^ and (RHC)_3_ and Beva; hydrogen bonding and hydrophobic bonding) between PZA and Beva because Beva is negatively charged, but the PZA nanoparticles are positively charged at pH 7.5 (Tang et al., 2022; Xu et al., 2024; Zhou et al., 2023).

### 3.3 The release and preservation of bioactivity of Beva

**Fig. 2:**
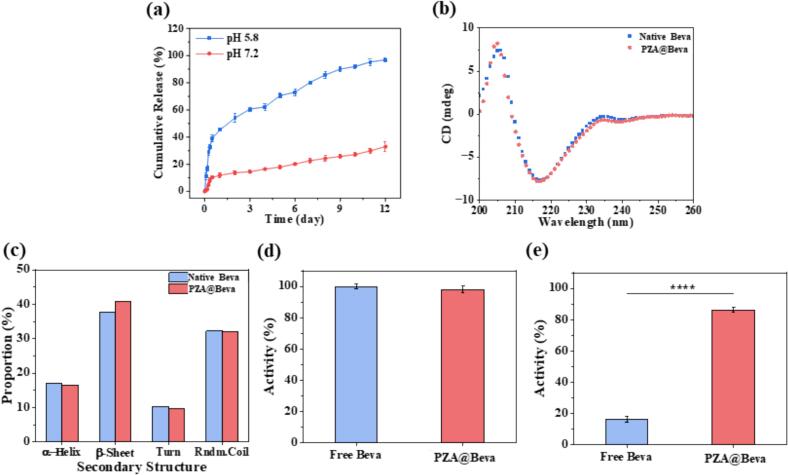
(a) The release behavior of PZA@Beva at pH 5.8 and 7.2. (b) The CD spectrum and (c) the proportion of secondary structure of free Beva and Beva released from PZA. (d) The bioactivity of free Beva and Beva released from PZA@Beva. (e) The bioactivity of Beva released by PZA@Beva after treating with protease K. Means (±S. E.) of n = 3 (at least) independent experiments.

This study explored the release behavior of PZA@Beva under different pH conditions. As shown in Fig. 2a, under acidic conditions (pH 5.8), PZA@Beva showed a burst release, followed by sustained release. Approximately 35 % of the Beva was released in the first 8 h. The drug release rate gradually slowed and the cumulative drug release was approximately 97 % after 12 days. However, at physiological pH (7.2), PZA@Beva showed persistent and slow-release behavior. On the 12th day, Beva released only about 37 % of Beva was released. Thus, it can be seen that PZA@Beva nanoparticles can achieve the effect of sustained and pH responsive drug release. This may be due to the strong coordination ability of Zn^2+^ with (RHC)_3_ in PZA nanoparticles in a neutral environment, but decreased in an acidic environment, which can easily lead to the disintegration of PZA particles.

The preservation of protein bioactivity is a major challenge in protein drug delivery research. The change in protein activity is usually accompanied by a change in secondary structure; therefore, circular dichroism (CD) can be used to detect whether the secondary structure changes before and after encapsulation of Beva. As shown in Fig. 2b, there was no significant difference between the CD spectra of Beva released by PZA and natural free Beva. Additionally, the proportions of the secondary structures of the two were compared using CDNN, a software associated with the CD instrument for protein secondary structure analysis. As shown in Fig. 2c, the proportion of β-fold of Beva released by PZA increased by 3.1 %, whereas the proportions of α-helices, β-corners, and random coils decreased slightly. This result suggests that the difference in the secondary structures may be due to the coordination between Zn^2+^ and Beva during the encapsulation of PZA. Therefore, we verified whether Beva activity was affected. A Beva ELISA kit was used for detection. As shown in Fig. 2d, after encapsulation and release by PZA, the activity of Beva still exceeded 98 %, indicating that the synthesis of PZA@Beva nanoparticles did not significantly affect the activity of Beva.

In addition, various proteases in the human body are major obstacles to the delivery of protein drugs. Proteases rapidly hydrolyze drugs, rendering them inactive. Therefore, to verify whether PZA was resistant to protease, protease K was incubated with free Beva and PZA@Beva at high temperature (55 °C), and its activity was detected using Beva ELISA kit. As shown in Fig. 2e, only 16 % of the free Beva activity was retained after proteinase K treatment, whereas the PZA@Beva nanoparticles retained 86 % of their activity. Thus, it can be seen that after PZA encapsulation, the activity of Beva was not only completely retained but also helped Beva resist the destruction of protease and achieve a better protein delivery effect.

### 3.4 Anti-angiogenesis in vitro

**Fig. 3:**
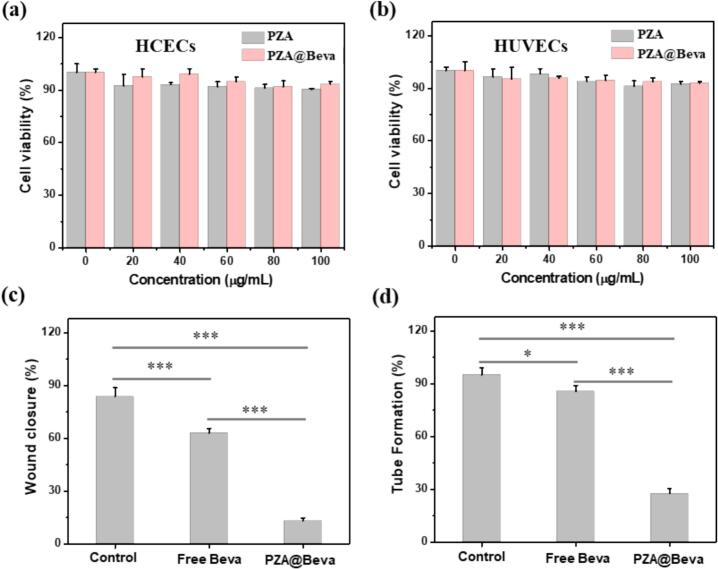
The cytotoxicity of PZA and PZA@Beva nanoparticles in (a) HCECs and (b) HUVECs for 24 h. (c) Statistical analysis of wound healing assays of Fig. S3. (d) Statistical results of tube formation assays on HUVECs after different treatments in Fig. S4. Means (±S. E.) of *n* = 3 (at least) independent experiments. *: *P* < 0.05; **: *P* < 0.01; ***: *P* < 0.001; ****: *P* < 0.0001.

The cytocompatibility of PZA and PZA@Beva was firstly investigated. The cytotoxicity test was performed using HCECs and HUVECs, and the results are shown in Fig. 3a-b. The concentration at 0 μg/mL (without adding any nanoparticles) was served as the positive control. When the concentration of PZA was increased to 100 μg/mL, the cell survival rate was above 90 % for both HCECs and HUVECs, indicating that PZA nanoparticles had good cytocompatibility. A concentration of 60 μg/mL PZA and PZA@Beva was used for subsequent cellular experiments to avoid controversy regarding the cytotoxicity of the materials. Vascular endothelial cell migration is an important process in angiogenesis, and its inhibition of endothelial cell migration prevents angiogenesis. In this study, HUVECs were used to determine the inhibitory effect of PZA@Beva on endothelial cell migration. As shown in Fig. S3, cells in the control and free Beva groups fused almost quickly, but there was no significant reduction in scratches in the PZA@Beva group. Quantitative analysis of the scratch area at 0 and 24 h after scratching (Fig. 3c) revealed that the percentage reduction in the scratch area in the PZA@Beva group was significantly smaller than that in the control and free Beva groups, indicating that PZA@Beva had a stronger ability to inhibit endothelial cell migration.

Tube formation by endothelial cells is a powerful basis for evaluating blood vessel formation in vitro. The HUVECs were used to observe the inhibitory effect of PZA@Beva on endothelial tube formation. As shown in Fig. S4, a mass of lumen structures appeared in the control group after the end of the experiment, whereas some lumen structures appeared in the free-Beva group and some cells showed budding or budding trends. However, no obvious budding trend was observed in the PZA@Beva group, and almost no lumen structures were formed. Statistical analysis (Fig. 3d) showed that the rate of cell tube formation in the PZA@Beva group was significantly lower than that in the control and free Beva groups, indicating that PZA@Beva nanoparticles had a stronger inhibitory effect on endothelial cell tube formation.

### 3.5 Cellular uptake, penetration in a 3D corneal model and occludin distribution of PZA@Beva

**Fig. 4:**
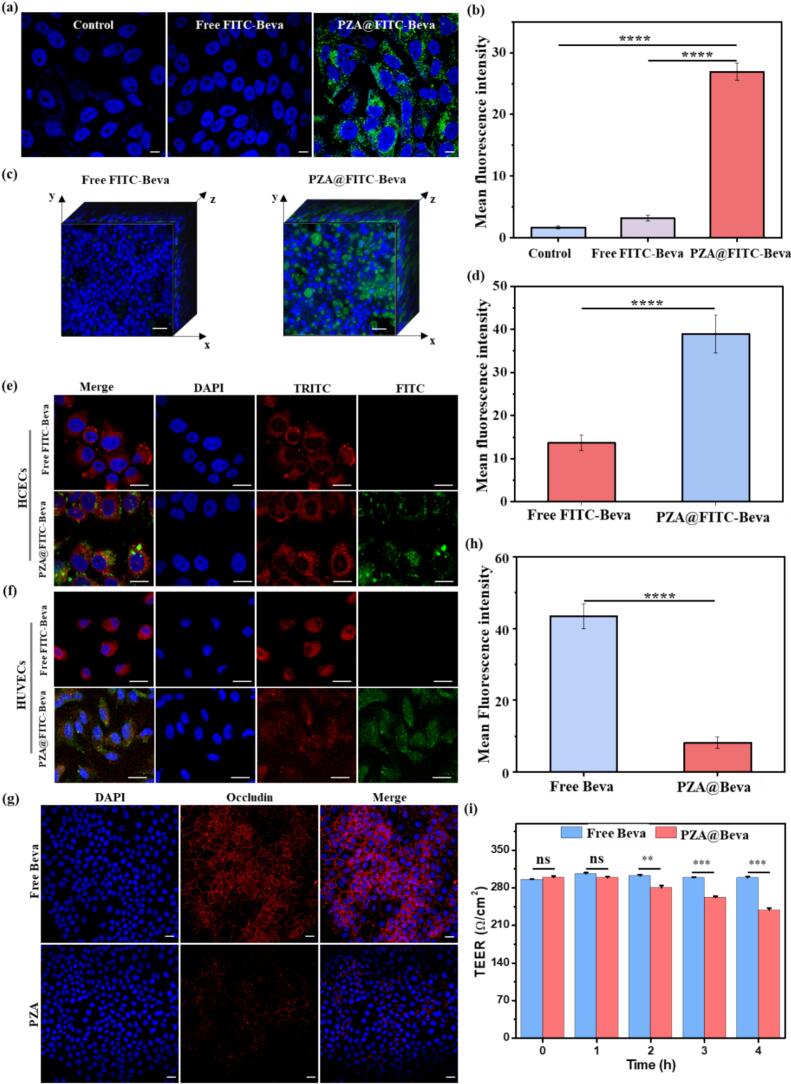
(a) The fluorescence microscopy images of HCEC after incubation with free FITC-Beva and PZA@FITC-Beva for 24 h. (b) Statistical results of mean fluorescence intensity of PZA@FITC-Beva and FITC-Beva from (a). (c) The fluorescence microscopy images of 3D multilayer corneal epithelial after incubating with PZA@FITC-Beva and free FITC-Beva for 24 h. (d) Statistical results of fluorescence intensity of PZA@FITC-Beva and FITC-Beva from (c). The fluorescence microscopy images of (e) HCEC and (f) HUVECs after incubation with PZA@FITC-Beva for 8 h. (g) The fluorescence microscopy images of occludin expression stained by immunofluorescence after incubating monolayered HCECs without (control) and with PZA for 6 h. (h) Statistical results of fluorescence intensity of occluding (red) from (g). (i) The TEER values of HUVECs cultured in transwell after incubating with free Beva or PZA@Beva. The green color suggests FITC-Beva, the blue color indicates nucleus, the red color in (e) and (f) indicate lysosome reporter and the occludin expression in (g). The scale bar in (a), (c), (e) and (f) were 50 μm, 200 μm in (e), and 25 μm in (h) and (i). Means (±S. E.) of *n* = 3 (at least) independent experiments. ****: *P* < 0.0001. (For interpretation of the references to color in this figure legend, the reader is referred to the web version of this article.)

The good cell uptake effect of the delivery carrier can improve the penetration of drugs on the ocular surface. Therefore, this study explored the PZA@Beva uptake ability of HCECs, and FITC-Beva was used as a fluorescence imaging drug. As shown in Fig. 4a, free Beva did not show obvious green fluorescence in cells, as in the control group, whereas a large number of green fluorescence signals were observed in HCECs in the PZA@FITC-Beva group. In the static analysis of the mean fluorescence intensity (Fig. 4b), there was an obvious difference between the free FITC-Beva and PZA@FITC-Beva groups, which was significantly lower than that in the control and free-Beva groups, suggesting that PZA nanoparticles could assist Beva uptake by HCECs. To verify the permeation effect of PZA@Beva nanoparticles in corneal tissue, the corneal 3D model was incubated with free FITC-Beva and PZA@FITC-Beva and then observed by laser confocal fluorescence microscopy. As shown in Fig. 4c, only a small amount of green fluorescence was observed on the surface of the corneal epithelium in the free FITC-Beva group. However, in the PZA@FITC-Beva nanoparticle group, a large amount of green fluorescence was observed in each structure of the corneal 3D model. The statistical results showed that the mean fluorescence intensity of PZA@FITC-Beva was stronger than that of FITC-Beva (Fig. 4d), indicating that PZA@FITC-Beva nanoparticles enhanced the corneal penetration of Beva into deeper corneal tissue. The lysosomal escape of the protein drug was examined by co-incubating PZA@FITC-Beva with HCECs and HUVECs for 8 h. As observed using Lyso-Tracker staining (Fig. 4e-f), very few yellow-colored dots were present in the merged image, indicating the successful escape of FITC-Beva from lysosomes. The calculated Pearson's coefficient, which serves as a measure of co-localization, was 0.18 and 0.21 for HCECs and HUVECs, respectively. The low Pearson's coefficient further supports the efficient lysosomal escape of PZA@FITC-Beva (Tang et al., 2022).

Occludin is an important transmembrane protein that forms tight junctions and plays a vital role in maintaining its integrity (Saitou et al., 2000). Its distribution in the adjacent epithelial space is an important parameter for evaluating epithelial cell permeability. HCECs were used to construct a monolayer epithelial barrier to evaluate the effects of PZA nanoparticles on occludin distribution. As shown in Fig. 4g, intercellular occludin was continuously distributed along the intercellular space in the control group, indicating that a dense and complete tight junction was formed between the HCECs. However, some occludin was scattered in HCECs treated with PZA nanoparticles (Fig. 4g), which was not as complete as that in the control group, indicating that PZA nanoparticles disrupted the integrity of tight junctions between HCEC. Statistical results showed that the mean fluorescence intensity of expressed occludin in PZA was lower than that without PZA treatment (Fig. 4h), indicating that PZA@Beva nanoparticles inhibited occludin expression. The effect of disrupting tight disruption was assessed by measuring the TEER values of HCECs cultured in Transwell plates after incubation with free Beva or PZA@Beva. As shown in Fig. 4i, the TEER value for free Beva remained approximately 300 Ω/cm^2^ and showed minimal change over time, indicating that tight junctions were formed in this model, and free Beva had no significant effect on their integrity. In contrast, the TEER value for PZA@Beva decreased sharply from 300 Ω/cm^2^ to 238 Ω/cm^2^, suggesting that PZA@Beva disrupted the tight junctions formed by HCECs.

Combining the above data, PZA was helpful for Beva to cross the corneal barrier into the corneal stroma through an intracellular pathway, enhanced penetration, and disruption of the tight junctions.

### 3.6 Long-term biosafety of PZA@Beva

**Fig. 5:**
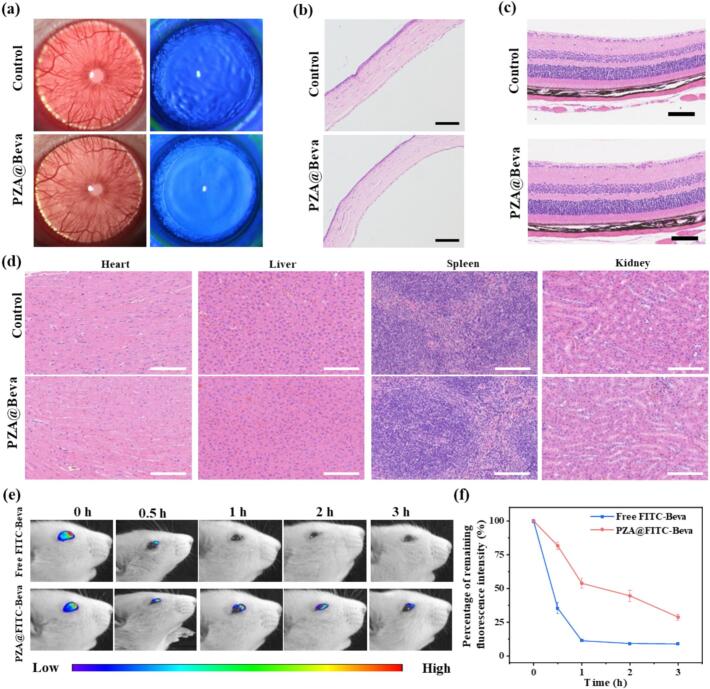
(a) The slit lamp images and the H&E staining images of (b) anterior segment, (c) retina, and (d) major organs (heart, liver, spleen, and kidney) of rats after 30 days successive topical administrated with normal saline (control) and PZA@Beva. (e) The representative in vivo fluorescent images of FITC-Beva, PZA@FITC-Beva at different time points on the ocular surface of rats. (f) Statistical analysis on the percentage of remaining fluorescence intensity from (e). The scale bar in (b) was 200 um and 100 μm in (c) and (d). Means (±S. E.) of *n* = 3 (at least) independent experiments.

The in vivo biocompatibility of PZA@Beva on the ocular surface was evaluated using a slit lamp and hematoxylin and eosin staining after 30 days of continuous eye treatment. The control group was topically administered normal saline and the experimental group was administered PZA@Beva nanoparticles. The results of slit lamp were shown in Fig. 5a, after 30 days, the rats in PZA@Beva group had clear corneas and no significant edema and epithelial cell defect, with no obvious abnormalities compared with the control group. As shown in Fig. 5b, H&E staining showed that after 30 days of continuous administration, the PZA@Beva group had no edema of the cornea, the structure was clear and intact, and no inflammatory cell infiltration was observed in any layer. As shown in Fig. 5c, no changes in the retinal morphology, including the retinal layers and their structures, were observed, and there were no signs of tissue damage or inflammation. Pathological examination of the major organs (heart, liver, spleen, and kidney) revealed no significant differences in organ structure and morphology between the PZA@Beva and control groups (Fig. 5d). Collectively, these findings support the safety of long-term ocular puncture with PZA@Beva.

### 3.7 Retention time of PZA@FITC-Beva on the ocular surface

A longer retention time is expected to improve the bioavailability of drugs on the ocular surface and to enhance drug penetration. Free FITC-Beva and PZA@FITC-Beva were dropped onto the ocular surface of normal rats, and a multi-mode in vivo imaging system was used for fluorescence imaging of the eyes of the rats. As shown in Fig. 5e, at 0.5 h, almost no obvious fluorescence retention was observed on the rat ocular surface. However, a large amount of fluorescence was retained on the ocular surface of rats in the PZA@FITC-Beva group, and the nanoparticles remained on the ocular surface for 3 h. Statistical analysis of the remaining percentage of fluorescence intensity (Fig. 5f) showed that only 35 % of fluorescence was retained in the free-Beva group at 0.5 h, while about 80 % fluorescence was still retained in the PZA@FITC-Beva group. In addition, 28 % of the drug was retained on the ocular surface after 3 h. PZA could significantly prolong the retention time of Beva on the ocular surface and play a crucial role in improving the effective utilization of the drug.

### 3.8 The effect of PZA@Beva on in vivo inhibiting neovascularization

**Fig. 6:**
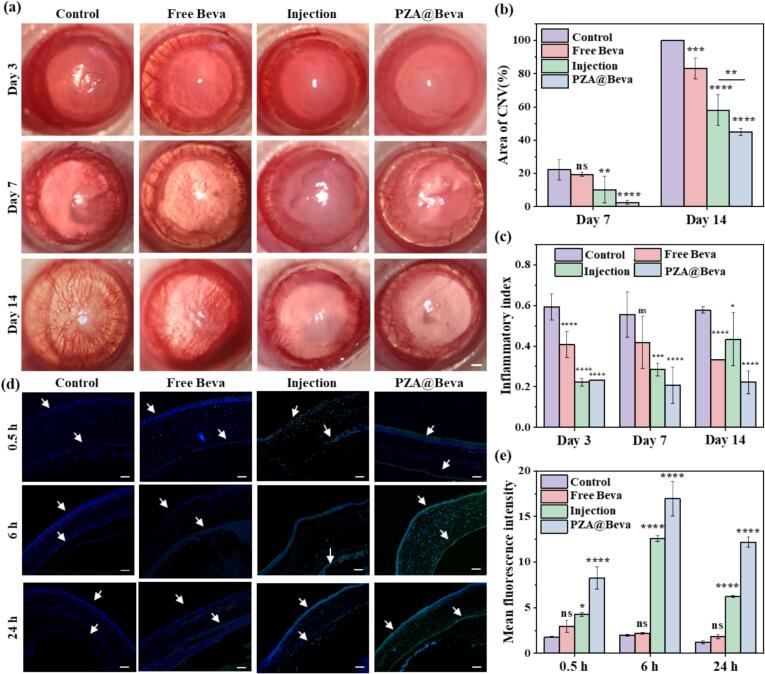
(a) Representative slit lamp anterior segment images of rats in each group (control group, Free Beva group, Injection and PZA@Beva group) at 3th,7th and 14th day; (b) CNV area ratio and (c) inflammatory index of all groups at 3th,7th and 14th day. (d) Fluorescence staining images of the FITC-Beva distribution of at 0.5 h, 6 h, and 24 h; The scale bar in (a) was 500 μm and in (d) was 20 μm. Means (±S. E.) of *n* = 3 (at least) independent experiments. *: *P* < 0.05; **: *P* < 0.01; ***: *P* < 0.001; ****: *P* < 0.0001.

In this study, a rat corneal neovascularization model was established using the alkali burn method and was divided into four groups: normal saline (control), free Beva, PZA@Beva, and subconjunctival injection of free Beva (a positive control used in clinical treatment for ocular delivery of Beva). The anterior segment was observed and recorded using slit lamps at 3, 7, and 14 days after administration (Fig. 6a). On the 3rd day, the corneas of each group had different degrees of edema, and no obvious neovascularization was observed. On the 7th day, corneal turbidity and edema were relieved, but new blood vessels were observed in the limbus of the rats in each group. After statistical analysis (Fig. 6b), the area of corneal neovascularization was the largest in the control group (22 %), followed by the free-Beva group (19 %), the injection group (10 %), and the PZA@Beva group (2 %). Compared to the control group, there was a significant difference in the PZA@Beva group (*P* < 0.01). On the 14th day, corneal edema disappeared; however, neovascularization was found in all areas of the cornea and grew to the center of the cornea (100 %) in the control group. The neovascularization areas were 83 %, 58 %, and 44 % in the free Beva, injection, and PZA@Beva groups, respectively. Compared to the control group, the results of each experimental group were significantly different. In conclusion, PZA@Beva had a better effect on inhibiting the growth of corneal neovascularization, and the therapeutic effect was significantly better than that of free Beva and the injection groups. The difference in results may be due to the better ocular surface retention and corneal permeability of PZA@Beva nanoparticles, which made it possible that after the drug was stopped on day 7, the remaining Beva in the corneal tissue of the PZA@Beva nanoparticle group could continue to be released from the nanoparticles to exert its inhibitory effect on the blood vessels.

The inflammatory index scale of Laira et al. was used to quantify the degree of inflammation in the eyes of the experimental rats (Laria et al., 1997). As shown in Fig. 6c, on 3rd and 7th day, the inflammation indices of each experimental group were lower than those of the control group were. On 14th day, the inflammatory index in the injection group was slightly higher than that in the free Beva group, which may be due to the subconjunctival injection procedure. Because subconjunctival injection is an invasive procedure, repeated injections may cause chemosis or aggravation of inflammation. Notably, the inflammation index of the PZA@Beva group was still significantly lower than that of the control and other experimental groups, which proved that PZA@Beva had a better therapeutic effect.

In this study, the ability of Beva to penetrate the corneal barrier was evaluated in each group by immunofluorescence staining. The results are shown in Fig. 6d. At each time point, no green fluorescence was observed in the corneal layers in the control group. At 0.5 h, green fluorescent signals of different intensities were observed on the surface of the corneal epithelial layer in the injection, and PZA@Beva groups, with the stronger being PZA@Beva. Over time, there was no obvious fluorescence on the corneal surface in the free-Beva group, a small amount of fluorescence signal was observed in the corneal endothelial layer in the injection group, and more green fluorescence signals were observed in all corneal layers in the PZA@Beva group. At 24 h, the green fluorescence signal was still observed in the corneal tissue of the injection and PZA@Beva groups, but the fluorescence intensity was slightly lower than that at 6 h. Statistical analysis (Fig. 6e) showed that the fluorescence signal in the PZA@Beva group was stronger than that in the free and injection groups at each time point, indicating that PZA@Beva nanoparticles had good corneal penetration and retention effects, which was beneficial for the better therapeutic effect of Beva.

**Fig. 7:**
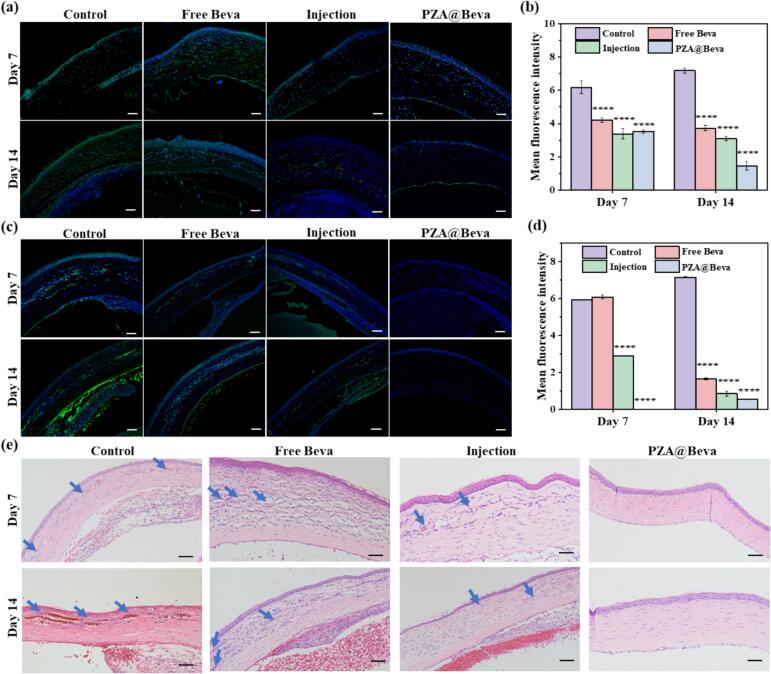
(a) The fluorescence images stained with VEGF-A. (b) Statistical analysis on the mean fluorescence intensity of rats' corneal pathological slices from (a). (c) The fluorescence images of expressed CD31. (d) statistical analysis on the mean fluorescence intensity of rats' corneal pathological slices from (c). (e) The corneal pathological sections stained with H&E of each group at day 7 and day 14. The blue arrow shows the location of the new blood vessels. The scale bars were 50 μm in (a) and (d). The scale bars were 20 μm in (e). Means (±S. E.) of n = 3 (at least) independent experiments. *: P < 0.05; **: P < 0.01; ***: P < 0.001; ****: P < 0.0001. (For interpretation of the references to color in this figure legend, the reader is referred to the web version of this article.)

Vascular endothelial growth factor (VEGF) is a highly specific vascular endothelial cell growth factor, which is the key substance to promote neovascularization (Nicholas and Mysore, 2021a). VEGF binds to its receptor to induce VEGF signal transduction and gene expression, regulate vascular permeability, and promote cell migration, proliferation, and survival. Beva specifically binds to VEGF and inhibit angiogenesis(Mukherji, 2010). In this study, the expression of VEGF-A in corneal tissues was detected using immunofluorescence, and the results are shown in Fig. 7a-b. On 7th day, more green fluorescence signals (indication of expression of VEGF-A) were observed in the corneal tissues of the control and free Beva groups, which were significantly higher than those in the injection and PZA@Beva groups. On the 14th day, except for the control group, the expression of VEGF-A in all experimental groups decreased, and the expression of PZA@Beva was the lowest, which was significantly different from that in the control group, indicating that PZA@Beva nanoparticles had a better therapeutic effect.

CD31, a platelet-endothelial cell adhesion molecule, is usually located in vascular endothelial cells and is mainly used to demonstrate the presence of endothelial cell tissue and evaluate angiogenesis (Caligiuri, 2020). Therefore, to further evaluate neovascularization, the expression and distribution of CD31 in the cornea were detected by immunofluorescence on 7th and 14th day. As shown in Fig. 7c, on the 7th day, a large amount of green lacunar fluorescence signals was observed in the corneal stroma in the control and free-Beva groups, and a small amount of green lacunar fluorescence signals was observed in the corneal stroma in the injection group, but no obvious green lacunar fluorescence signals were observed in the PZA@Beva group. On the 14th day, an increase in green fluorescence was detected in the control and free Beva groups; in contrast, almost no fluorescence was observed in the injection and PZA@Beva groups. Further quantitative fluorescence statistics (Fig. 7d) showed that during treatment, the PZA@Beva group had the lowest expression of CD31.

Histological analyses were performed in this study. As shown in Fig. 7e, on 7th and 14th day, the control and free Beva groups showed corneal edema, disorganized tissue structure, increased inflammatory cell infiltration, and the presence of vascular cavities in the stromal layer (blue arrows). On the 7th day, the injection group showed obvious corneal edema accompanied by inflammatory cell infiltration and a small amount of vascular lumen formation. Corneal edema and inflammatory cell infiltration were alleviated and vascular cavities were still observed in the stroma on 14th day. The greatest curative effect was observed in the PZA@Beva group. At 7th or 14th day, a complete corneal structure was observed without obvious edema or inflammatory cell infiltration. These results were consistent with the CD31 staining results, indicating that PZA@Beva nanoparticles had better inhibited corneal neovascularization.

## 4 Discussion

Subconjunctival injections are the predominant method of protein delivery for the treatment of ocular surface diseases in clinical practice (Rieke et al., 2010). The subconjunctival injection technique is associated with a lower risk profile than the posterior ocular injection; however, the potential for infection remains (Yoon et al., 2009). Furthermore, the psychological stress associated with subconjunctival injections can result in patient resistance. Although eye drops offer a high level of safety and patient compliance, their low delivery efficiency has been a subject of criticism (Lakhani et al., 2018). In the CNV model, PZA@Beva eye drops demonstrated a therapeutic effect comparable to that of subconjunctival injection administration of free Beva (Fig. 6, Fig. 7), indicating that PZA may be an effective solution to overcome the low delivery efficiency of traditional eye drops.

The reasons for the ability of PZA to enhance the efficiency of protein delivery may be multifactorial, including prolonged drug retention on the ocular surface and improved penetration of the particles in the eye. The positive charge of PZA enables it to attach to the ocular surface via electrostatic interactions with negatively charged proteins and polysaccharides (Wang et al., 2020). Furthermore, the sulfhydryl functionality of PZA enables the formation of covalent disulfide bonds with sulfhydryl groups in mucin, thereby extending its retention time on the ocular surface (Zhang et al., 2018; Armengol and Laffleur, 2021). With regard to enhancing particle penetration on the ocular surface, PZA not only exhibited favorable endocytosis and permeability in a multilayer cell model, but also, more significantly, demonstrated the ability to open tight junctions between epithelial cells (Fig. 4). Tight junctions are a significant barrier at the ocular surface that impede the penetration of exogenous pollutants. Loosening of these junctions facilitates penetration of PZA particles into the ocular surface.

Furthermore, the characteristics of PZA in protein encapsulation represent a significant contributing factor to the effective treatment of CNV. An increased drug-carrying capacity is conducive to the rapid attainment of a therapeutic drug concentration, while maintaining a similar penetration efficiency. Compared with the majority of previously documented carriers (DeFrates et al., 2018), the protein drug-carrying capacity of PZA was markedly superior, reaching an EE% of 92.7 % and an LC% of 55.8 %. Second, the mild processing conditions of PZA confer an intrinsic advantage for the maintenance of protein function. PZA protects the encapsulated proteins from protease degradation, thereby ensuring the activity of the protein drug during delivery.

The manner in which PZA is constructed allows the encapsulation of other proteins and small-molecule drugs (Huang et al., 2022). Given the high delivery efficiency of PZA, it may have significant applications in the treatment of other ocular surface diseases through non-invasive interventions. In the context of ocular administration, it is essential to consider the stability of zinc ions and the potential physiological toxicity associated with long-term accumulation, despite the low content of zinc ions in PZA eye drops. In particular, the eye has a relatively closed circulatory system, which may exacerbate the physiological toxicity of zinc ions (Matsumoto et al., 2017; Greaves and Perkins, 1952). Additionally, PZA disrupts the tight link barrier, which may facilitate the invasion of foreign pathogens and pose a potential risk (Naeem et al., 2011). Further evaluation is required to ascertain the extent of tight-link disruption caused by PZA and the process of tight-link repair, particularly in the context of prolonged ocular administration. Although the available data suggest that PZA does not exhibit visible physiological toxicity (Fig. 3a-b, and Fig. 5a-e), further evaluation of its biosafety is necessary before conducting clinical trials.

## 5 Conclusion

In conclusion, we successfully synthesized a positively charged and uniformly sized nanocarrier (PZA) with a high encapsulation efficiency and pH-dependent drug release characteristics for Beva. In vitro experimental data have shown that PZA can efficiently open tight junctions, rapidly endocytosed by cells, and effectively inhibit cell migration and angiogenesis. In addition, PZA retained the biological activity of the encapsulated Beva and protected it from protease damage. In vivo experiments demonstrated that PZA@Beva greatly prolonged the residence time of the drug on the ocular surface, showed the ability to overcome the ocular surface biological barrier, and produced surprisingly good results in a corneal neovascularization model, even better than the better subconjunctival injections frequently used in clinical practice. This study provides a new approach for non-invasive delivery of protein drugs for the treatment of anti-ocular front-end diseases.

## Author contribution

Y. Tong, and S. Zhou investigated and designed the study. Y. Tong, S. Zhou, X. Jin, and M. Yu performed most experiments. Y. Tong, S. Zhou, and Y. Guo analyzed data and software application. C. Feng, Y. Guo, and X. Jin had intellectual contributions. Y. Tong and S. Zhou wrote the original manuscript. X. Zan and H. Chen revised the manuscript. X. Zan and J. Li supervised this work. H. Chen and J. Li acquired the funding. All authors have read and approved the final manuscript for submission.

## Declaration of competing interest

The authors declare that they have no known competing financial interests or personal relationships that could have appeared to influence the work reported in this paper.

## Data Availability

Data will be made available on request.

## Appendix A Supplementary data

Supplementary material Image 1
