# Supplementary material for: Co-assembling de novo designed peptide with high-payload drug protein for noninvasive treatment of corneal neovascularization
Source: Int J Pharm X. 2025 Sep 28;10:100410. doi: 10.1016/j.ijpx.2025.100410 (PMC12528919; doi:10.1016/j.ijpx.2025.100410)
Supplement: Supplementary file 1 — Supplementary material [file mmc1.docx]

**Supplementary material**

**Co-assembling de novo Designed Peptide with High-payload Drug Protein for Noninvasive Treatment of Corneal Neovascularization**

*Yuhua Tong ^a ,b 1^, Sijie Zhou ^a, c 1^, Yongjie Guo ^d^, Xiaoli Jin ^a, e, f^, Meiting Yu ^a, b^, Chunyun Feng ^a, b^, Hao Chen ^a, e, f *^, Xingjie Zan ^g *^, and Jinyang Li ^a, e, f *^*

^a^ National Clinical Research Center for Ocular Diseases, Eye Hospital, Wenzhou Medical University , Wenzhou, 325027, China.

^b^ Department of Ophthalmology, The Quzhou Affiliated Hospital of Wenzhou Medical University, Quzhou People's Hospital , Quzho , 324000, Zhejiang, China

^c^ Department of Ophthalmology, Second Affiliated Hospital of Chongqing Medical University, 400010 Chongqing, China

^d^ No. 906 Hospital of People's Liberation Army, Wenzhou 325000, Zhejiang, China

^e^ National Engineering Research Center of Ophthalmology and Optometry, Eye Hospital, Wenzhou Medical University, Wenzhou, 325027, China

^f^ State Key Laboratory of Eye Health , Eye Hospital , Wenzhou Medical University , Wenzhou , 325027, China

^g^ Wenzhou Institute, University of Chinese Academy of Sciences, Wenzhou 325000, Zhejiang, China

*^1^* Equal contribution author

* Corresponding author. E-mail: zanxj@ucas.ac.cn (X. Zan); Lijinyang@eye.ac.cn (J. Li); Chenhao823@mail.eye.ac.cn (H. Chen)

**Figure S1.** The standard curve of FITC-Beva (absorbance intensity at 495 nm under UV-vis plotted against the concentration of FITC-Beva), with concentration range of 5-600 ug/mL. For each point, the average value of tri-replicates with standard error was used for the fitting. The limits of detection (LOD) and quantification (LOQ) are 1.9 ug/mL and 6.25 ug/mL, respectively. The insert equation was the linear relationship between intensity and concentration of FITC-Beva, and the inserted number R^2^=0.99 was the fitting co-efficiency.


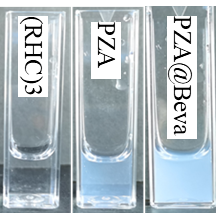
**Figure S2.** The photo images for the states of (RHC)_3_, PZA and PZA@Beva solutions.


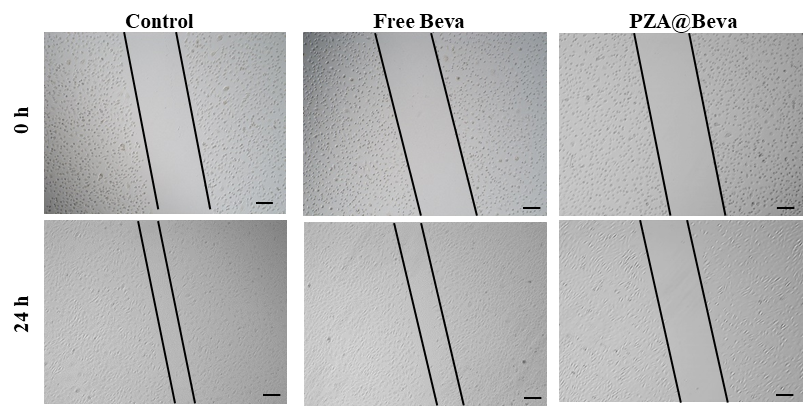
**Figure S3.** The representative photographs of the scratch at 0 h and 24 h after the generation of wounds of HUVECs. Scale bar: 50 μm.

**
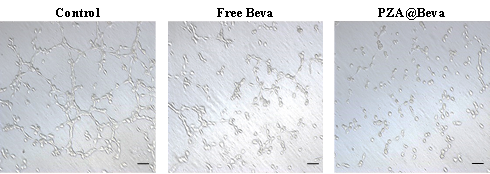
Figure S4.** Representative images of tube formation of HUVECs. Scale bar: 100 μm.

**Table S1** Effects of Beva concentration on the size, PDI and zeta potential of PZA@Beva nanoparticles.

| **Beva concentration (mg/mL)** | **Size (nm)** | **PDI** | **Zeta potential (mV)** |
| --- | --- | --- | --- |
| 0 | 104.21±5.49 | 0.21±0.03 | 25.72±3.99 |
| 0.5 | 110.02±6.42 | 0.20±0.04 | 24.61±4.15 |
| 1 | 132.01±3.28 | 0.21±0.06 | 24.33±4.85 |
| 2 | 162.51±4.36 | 0.25±0.07 | 23.93±4.35 |
| 3 | 195.12±10.72 | 0.32±0.14 | 23.72±4.32 |
| 4 | 280.03±18.38 | 0.35±0.18 | 23.51±4.26 |
| 5 | 340.03±20.51 | 0.43±0.27 | 23.22±4.21 |
| 6 | 330.02±25.33 | 0.57±0.33 | 24.2a±4.27 |
